# CARDIAC INVOLVEMENT IN PATIENTS OF SYSTEMIC SCLEROSIS

**DOI:** 10.4103/0019-5154.44801

**Published:** 2008

**Authors:** Qazi Masood Ahmad, Iffat Hassan Shah, Qazi Nauman, Farah Sameem, MA Kamili

**Affiliations:** *From the Department of Dermatology, STD and Leprosy and Department of Internal Medicine, Government Medical College, Srinagar Kashmir (Jammu and Kashmir), India*

**Keywords:** *Cutaneous metastases*, *melanoma*, *cardiac involvement*, *systemic sclerosis*, *ventricular hypertrophy*

## Abstract

**Background:**

Systemic sclerosis is a multi-systemic autoimmune disorder. Cardiac involvement by the disease, although not included in the diagnostic criteria, may be seen either clinically, histologically or may be revealed by various investigative modalities.

**Purpose:**

To see the profile of cardiac involvement in patients of systemic sclerosis.

**Materials and Methods:**

Forty-seven patients of systemic sclerosis were included in the study. After taking a complete history and doing a detailed physical examination, the patients were submitted to electrocardiogram ECG (all leads), echocardiography and x-ray chest. Furst's organ indices scoring system for cardiac involvement was followed.

**Findings:**

Forty-seven patients of systemic sclerosis were included in the study. Five females gave a history of palpitations. A loud pulmonic heart sound was heard in 1. Arrhythmias were observed in 5 patients. Significantly, echocardiography revealed valvular involvement in 5 patients. Left ventricular hypertrophy was seen in 2 patients.

**Conclusions:**

In our patients, cardiac involvement was rare. In contrast to other studies, valvular involvement was a prominent feature.

**Limitations:**

Complete evaluation for arrhythmias with 24-h Holter monitor was not used

## Introduction

Systemic sclerosis is a multi-systemic autoimmune disorder affecting predominantly the skin, lungs, gut and kidneys. The criteria to be fulfilled for labeling a patient as a case of systemic sclerosis (established by the Sub-committee for Scleroderma Criteria of the American Rheumatology Association) includes one major criteria, i.e., sclerosis of skin proximal to the digits including the face, limbs, neck or trunk and/or two or more minor criteria, i.e., (1) Sclerodactyly (2) Digital pitted scarring and (3) Bilateral Lower zone (basal) pulmonary fibrosis.^1^ Cardiac involvement by the disease, although not included in the diagnostic criteria, may be seen either clinically, histologically or may be revealed by various investigative modalities. Furst *et al.,* developed indices of organ involvement for patients with systemic sclerosis for initial assessment and determining effectiveness of therapeutic intervention.^2^ For cardiological assessment, three indices were included: cardiac, left heart and right heart.

## Materials and Methods

Forty-seven patients of systemic sclerosis admitted in the in-patient wing of the Department of Dermatology, STD and Leprosy SMHS Hospital (Associated teaching hospital of Government Medical College Srinagar) during 1998–2005 were included in the study. The diagnosis of systemic sclerosis was made on the basis of the ARA criteria. A complete history was taken, especially regarding the presence of chest pain, dyspnea and palpitations. A detailed physical examination was done, including recording the weight, pulse, blood pressure, examination of chest, cardiovascular system (especially the heart rate and the heart sounds) and abdomen. This was followed by a meticulous cutaneous examination and Furst's skin scoring. The patients were submitted to a battery of investigations, including electrocardiogram ECG (all leads) to examine the rate, rhythm, axis, PR interval, P- and T- wave changes, echocardiography to examine the valvular involvement including vegetations if any, atrial and ventricular involvement, coronary reserve and radiograph of Chest for observing the cardiac size.

Furst's organ indices scoring for complete cardiological assessment was done with the following parameters:

### 1. Cardiac findings

Cardiomegaly on x-ray chest or moderate-to-large pericardial effusion on echocardiogram – absent: 0; present: 1.5

S ymptomatic congestive heart failure – absent: 0; present: 2

Symptoms of arrhythmias (palpitations, syncope, fainting associated with ventricular tachycardia or >5 premature ventricular contractions) – absent: 0; present: 1.5

Echocardiographic score (1 point for right ventricular dilatation or hypertrophy and 1 point for left ventricular dilatation or hypertrophy) 0–2

### 2. Right heart findings

Points:

Accentuated second pulmonic sound on physical examination – absent: 0; present: 2

Right ventricular hypertrophy right axis deviation or right atrial enlargement on ECG – absent: 0; present: 2

Right bundle branch block on ECG – absent: 0; present: 2

Maximum right heart score = 6

### 3. Left heart

Left ventricular hypertrophy, left axis deviation or left atrial enlargement on ECG – absent: 0; present: 2

Left bundle branch block on ECG – absent: 0; present: 2

Intraventricular conduction delay on EKG – absent: 0; present: 2

M yocardial infarction by history or on ECG – absent: 0; Present: 2

Maximum left heart score = 8

## Results

Forty-seven patients of systemic sclerosis were included in the study. They included 45 females and 2 males (F:M ratio of 23:1). The age range of patients was 18–60 years (average age of presentation: 40 years). The duration of disease prior to presentation ranged from 1 month to 25 years. Amongst the associated medical conditions were diabetes (in 1 patient) and hypertension (in 2). Of these 47 patients, none showed cardiomegaly on chest x-ray. None had pericardial involvement. No patient was symptomatic for congestive cardiac failure. Five females gave a history of palpitations. Left ventricular hypertrophy was observed in 2 patients and right ventricular dilatation in 3. Evidence of Pulmonary Arterial Hypertension – loud second pulmonic sound on auscultation and right atrial enlargement (P pulmonale) on ECG was observed in 5 patients. Myocardial infarction was not observed in any patient.

Arrhythmias including ventricular premature contractions and evidence of bradycardia and left anterior or posterior hemiblock or left bundle branch block on ECG were observed in 5 patients. None of the patients had paroxysmal atrial tachycardia, atrial flutter or fibrillation. Significantly, echocardiography revealed valvular abnormalities in the form of mitral regurgitation (2 patients), mitral stenosis (2 patients), aortic regurgitation (2 patients), and Patent ductus arteriosus (1 patient).

## Discussion

Cardiac involvement^3^,^4^ in systemic sclerosis is usually asymptomatic. Dyspnea may be present but pain in chest is not a prominent feature. The heart may show no changes macroscopically, but widespread histological changes may be observed in the form of focal or diffuse fibrosis in the myocardium, pericarditis with effusion, Libman-Sacks type of nonbacterial endocarditis of the mitral or tricuspid valve, aortic panaortitis and valvulitis. The coronaries are usually patent, although the smaller arteries or arterioles show thickening of the walls.^1^

The resting ECG is abnormal in 50% of the cases.^5^ Abnormalities of rhythm include paroxysmal atrial tachycardia, atrial flutter or fibrillation. The conduction system is relatively spared. Partial or complete heart block is not uncommon. ECG may show bifid P- and T- wave changes indicating atrial and ventricular myocardial involvement. Mitral valve prolapse is observed with other valvular abnormalities being rare.^6^

In our patients, cardiac involvement was rare. In contrast to other studies, valvular involvement was the prominent feature with mitral stenosis, mitral regurgitation, patent ductus arteriosus and aortic regurgitation seen, and no case of mitral valve prolapse detected. Significantly, one of our patients – a young female with severe vascular involvement in the form of Raynaud's recalcitrant to vasodilators – also complained of chest pain, and she died at home during harsh winter season, thereby leading one to conjecture an adverse cardiac event as a cause of death. (Cold-induced change in cardiac activity is known to occur.^5^)
